# Specialties preference by gender among medical students at Sefako Makgatho Health Sciences University, South Africa

**DOI:** 10.4102/safp.v66i1.5858

**Published:** 2024-04-23

**Authors:** Andiswa Pooe, Samuel T. Ntuli, Sizwe Masango, Aqila Rab, Thiambi Mudau, Pollet M. Mantsho, Sifundo Mtshali

**Affiliations:** 1 Department of Haematological Pathology, Faculty of Medicine, Sefako Makgatho Health Sciences, Pretoria, South Africa; 2 Department of Statistical Sciences, Faculty of Science and Technology, Sefako Makgatho Health Sciences, Pretoria, South Africa

**Keywords:** medical students, gender differences, career preference, speciality choice

## Abstract

**Background:**

To determine the speciality preferences and the gender differences in the choice of speciality among medical students at Sefako Makgatho Health Sciences University, South Africa.

**Methods:**

This cross-sectional study was conducted among fourth- to sixth-year medical students. A structured self-administered questionnaire was used to collect the data. Data analysis was performed using STATA version 16 (StataCorp, College Station, TX, United States).

**Results:**

A total of 174 students participated (response rate of 74%). Their median age was 23 years with interquartile range of 2 years. More than half (57%) were females. About 83% had no previous qualifications. Most (89%) have shown interest in pursuing specialist training. Surgery, obstetrics and gynaecology and internal medicine were the most selected specialities, while family medicine, ophthalmology, forensic medicine, public health medicine, ear, nose and throat, and accident and emergency medicine were the least preferred. Males were more likely interested in surgery and internal medicine, while females preferred obstetrics and gynaecology.

**Conclusion:**

The majority of the medical students intends to pursue their postgraduate medical training. Even though the results were not statistically significant, there are gender differences in speciality preferences. There is a need to develop and implement career guidance and recruitment plans to deal with specialities with poor recruitment and gender imbalance.

**Contribution:**

To deal with specialties with poor and gender imbalance, career guidance and recruitment plans must be developed and implemented.

## Introduction

Globally, healthcare systems are faced with many challenges, including rapid growth in the aging population, emerging new illnesses and changes in patterns in the existing ones. This is aggravated by the severe shortage,^1,2,3,4^ migration^5,6^ and uneven distribution of the human resources for health both in areas of speciality^7,8,9^ and geographical location.^10^ Medical schools comprise several specialities and have played a critical role in trying to address some of these challenges.^11,12,13^ However, career choice by medical students has been problematic, because certain specialities are less preferred. For years, the selection of specialist training among medical students has been an issue that attracted the interest of researchers worldwide. There are many studies in developing countries on medical students’ career aspirations. Most of these studies found that greater proportions of medical students are interested in pursuing specialist training post-graduation. As described in studies conducted in Pakistan^14^ and India,^9^ 96.5% and 96.7% of the medical students intended to pursue medical postgraduate training, respectively.

In sub-Saharan African countries, between 85.7% and 91.2% of the medical students were also found to be interested in pursuing postgraduate medical training.^15,16,17^ An earlier study in South Africa (SA) found that 47%^18^ and 85.8%^19^ of the medical students intended to specialise and made a definite career choice. The most selected career aspiration for medical specialisation includes surgery, internal medicine, obstetrics or gynaecology and paediatrics.^9,16,17,18,19,20,21,22,23,24^ Azu et al.^19^ stated that speciality preference by medical students is constantly changing over time and graduates decides to choose certain specialities over others, because of various factors such as plans to have a family, acceptable hours of practice, intellectual challenge, opportunities for health promotion and prevention, working with new technology and continuity of patient contact.^18^ This situation highlights the need for continuous evaluation of career choice to promote equitable distribution of medical specialists among different career domains to meet community needs.

Gender variant in the choice of specialities is also important for continuous evaluation, with male medical students shown to prefer surgery over females.^14,17,20,22,24,25,26^ Several studies have shown that females selected internal medicine as compared with males,^14,20,22,23,27^ whereas others found no difference between males and females in choosing internal medicine as a career speciality.^17,25^ Some studies reported that females significantly preferred obstetrics and gynaecology and paediatrics more than males.^26^

Numerous factors have been found that influence medical students’ choices for a specific speciality, such as inspiration during clinical practice, financial reward, dedication to the field, benefits to a patient, the department is organised, possession of competency needed for the speciality, mentorship, the influence of peer and lecturers, lecturers friendly, offered good advice and related well with students and personal interest.^16,19,23,24^

An understanding of the career prospects of medical students may provide essential information helpful for the planning of postgraduate education programmes and addressing the shortage of medical students in certain specialities to provide optimal health needs for the population. Globally, studies have been conducted and show a gender difference in speciality choice.^14,18,20,22,25,26^ Few studies on career preferences within the South African context have been conducted in medical schools among students in various academic levels.^18,19,28,29^ Most of these studies have been conducted for long period, and such research is needed again for an update.^18,19,29^ In our institution, no studies investigated career preferences and which specialities are mostly preferred. Moreover, our setting lacks information on gender differences regarding speciality preferences. Therefore, our study aimed to determine medical students’ speciality choice at the time when they are about to complete training and assess the importance of sex, age and academic level of study in choosing a medical speciality.

## Methods

### Study design and setting

This cross-sectional descriptive study was carried out from 15 July 2022 to 19 August 2022 at Sefako Makgatho Health Sciences University, which is one of the three medical schools in Gauteng province, South Africa. The institution is a non-profit public higher education consisting of five schools: the School of Medicine, Pharmacy, Science and Technology, Healthcare Science and Oral Health Sciences. During the 2022 academic year, there were 886 registered medical students, of which 275 in the fourth academic year, 267 in the fifth academic year and 344 in the sixth academic year.

### Study population

The study population was registered medical students at Sefako Makgatho Health Sciences University in their fourth, fifth and sixth year who gave consent to participate in the study.

### Sample size and sampling technique

The minimum sample size required for the study was 122, which was calculated using Roasoft, an online sample size calculator. The following information was used to calculated sample size: a 95% confidence interval, a 5% margin of error, 886 registered 4th, 5th and 6th medical students and 89.8% a proportion of medical students’ in SA intended to specialise after graduation.^29^ The sample size was distributed proportionally to the population size of the medical students per academic level of study, and a simple random sampling technique was used to select the study participants.

### Data collection

The questionnaire was developed by the researchers using relevant literature.^18,19,28,29^ The tool collected information on socio-demographic characteristics such as age, gender, previous qualification, intention to pursue postgraduate medical training and their speciality of choice. To determine the participants choice of specialty, the following question was asked ‘What is your planned future speciality?’ The tool was given to the panel of experts in research to evaluate its face and content validity (i.e. appearance, clearness and comprehensibility of the questions) and pre-tested on a convenience sample of medical students.

### Data analysis

Data were analysed using the statistical programme STATA version 16.0 (StataCorp LLC, College Station, TX, United States). All normally distributed results were described using means and standard deviations, while those that were not were described using medians and interquartile ranges, based on the Shapiro-Wilk test findings. Categorical variables were presented as frequencies and percentages. Comparison between groups was performed using the Fisher exact test. A *p*-value of less than 0.05 was considered statistically significant.

### Ethical considerations

This was a group research project for fourth-year medical students, thus, the ethical clearance to conduct this study was obtained from the Sefako Makgatho Health Sciences University Research Ethics Committee (REF: SMUREC/M/146/2022: UG).

Permission to conduct the study was sought from the School of Medicine where data were collected. The participants were informed about the aim and objectives of the study before completing the informed consent form. Participants were also informed that participation is voluntary and that they could withdraw from the study at any time.

## Results

### Demographic characteristics

A total of 174 medical students participated in this study (response rate of 74%). Slightly more than half (51%, *n* = 89) of the participants were in the fourth year (27%, *n* = 47), fifth year and (22%, *n* = 38) in the sixth year. The median age of the participants was 23 years with interquartile range of 2 years. More than two-thirds (75%) of the participants were less than 25 years old, and 57% were females. The majority (83%) had no previous qualifications. Table 1 shows a detailed description of the participants per academic level of study.

**TABLE 1:** Demographic characteristics of the participants.

Variable	*n*	%	Academic year of study					
			4th (*n* = 89)		5th (*n* = 47)		6th (*n* = 38)	
			*n*	%	*n*	%	*n*	%
**Age**								
< 25	131	75	73	82	38	81	20	53
25–29	41	24	16	18	9	19	16	42
30+	2	1	-	-	2	5	-	-
**Gender**								
Males	72	41	39	44	17	36	16	42
Females	102	59	50	56	30	64	22	58
**Had the previous qualification**								
Yes	29	17	18	20	3	6	8	21
No	145	83	71	80	44	94	30	79

The participants were asked about their preferred medical speciality for their future careers. Most (89%, *n* = 154) have shown interest in pursuing medical specialist training in different fields and 20 (11%) had not yet decided on their future medical career speciality. As shown in Table 2, there was no statistically significant relationship between the participants’ demographic characteristics and preference for pursuing medical specialist training (*p* > 0.05).

**TABLE 2:** Association between students’ intention to pursue specialist training and demographics.

Variable	Intended to pursue specialist training				*p*-value
	Yes		No		
	*n*	%	*n*	%
**Age**					0.275
< 25	118	90	13	10	
≥ 25	36	84	7	16	
**Gender**					0.894
Males	64	89	8	11	
Females	90	88	12	12	
**Had the previous qualification**					0.671
Yes	25	86	4	14	
No	129	89	16	11	
**Level of study**					0.599
4th	79	89	10	11	
5th	40	85	7	15	
6th	35	92	3	8	

**FIGURE 1:**
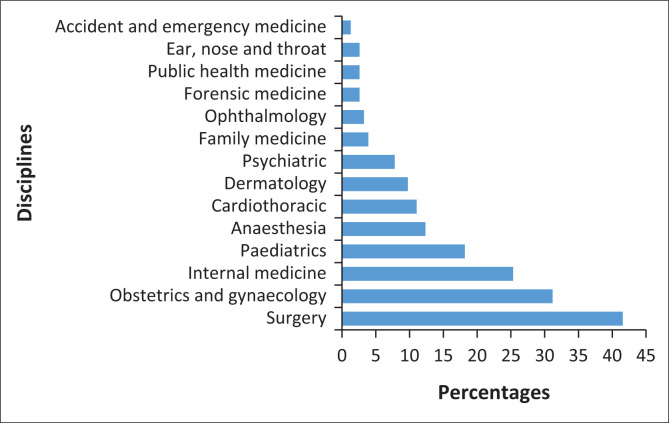
Students’ speciality of choice (*n* = 154).

Of the 154 medical students who indicated that they are interested to pursue specialist training, slightly more than half (57%) choose two or more specialities (i.e. 38% chose two, 14% chose three and 5% chose four). The three most common preferred specialities were surgery, obstetrics and gynaecology and internal medicine. Family medicine, ophthalmology, forensic medicine, public health medicine, ear, nose and throat, and accident and emergency medicine were the least preferred choices (Figure 1).

**FIGURE 2:**
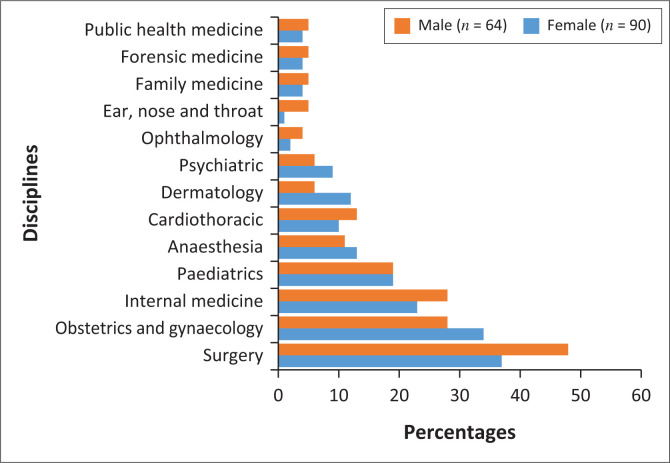
Description of choice of speciality by sex (*n* = 154).

Figure 2 illustrates speciality preference by gender. The results revealed that males tend to show more interest than females in surgery (48% versus 37%, *p* = 0.184), internal medicine (28% versus 23%, *p* = 0.574), cardiothoracic (13% versus 10%, *p* = 0.795), accident and emergency medicine (3% versus 0%, *p* = 0.171) and ophthalmology (4% versus 2%, *p* = 0.650).

Whereas females had more interest in obstetrics and gynaecology (34% versus 28%, *p* = 0.484), anaesthesia (13% versus 11%, *p* = 0.805), dermatology (12% versus 6%, *p* = 0.276) and psychiatry (9% versus 6%, *p* = 0.762); however, both genders equally preferred paediatrics (19% versus 19%, *p* = 1.000). None of the males had chosen family medicine (0% versus 7%, *p* = 0.042), forensic medicine (0% versus 4%, *p* = 0.142) and public health medicine (0% versus 4%, *p* = 0.142).

## Discussion

This study aimed to evaluate the proportion of medical students who intend to specialise after graduation and determine the most preferred speciality career at Sefako Makgatho Health Sciences University. From our study, the proportion of students who intended to specialise after graduation was 89%, which is similar to 89.5% reported in Nigeria,^17^ 89.8% in SA^29^ and 87.9% in Botswana^30^ but slightly higher than 85.7% in Ethiopia^16^ and 83% in SA.^19^ Our finding is, however, lower than 91.2% reported in six sub-Saharan African countries,^15^ 96.5% in Pakistan^14^ and 96.7% in India.^9^ The finding of our study is encouraging, as a higher number of medical students are considering continuing with post-graduate training, which is an indication that the shortage of specialists in the country can be lessened.

In our study, more than half (53%) of the students selected two or more specialities as a future career, which indicates that they did not know what their primary discipline might be. The reason for this could be that students commence their medical training having little or no career guidance, which implies that they enter medical school without any prior knowledge of different specialities making room for uncertainties.^19^ Another interesting finding reported in the current study is that 12% of the students did not yet decide on their future field of speciality. This finding is similar to the 11.2% reported in Ethiopia^16^ and 10.5% in Nigeria,^17^ but lower than the rate of 22.4% found in Jordan.^23^ Our finding is, however, higher than the rate of 7.1% reported in Iran,^7^ 3.5% in Pakistan^14^ and 4% in Nelson R. Mandela School of Medicine in South Africa.^28^ The possible reason for this discrepancy could be that the population of interest in these studies were from different academic levels and had not yet rotated to various specialities; hence they are unable to decide.

Our study findings revealed that the two most favoured specialities after graduation were surgery and obstetrics and gynaecology, which agrees with the finding of a study in Uganda.^22^ In contrast, other studies from Kenya,^20^ Syria,^21^ India^9^ and Ethiopia^16^ found that surgical fields in combination with internal medicine were the two most career preferences of medical students, while others reported the combination of surgery and paediatrics in Nigeria.^17^ Evidently, internal medicine and surgery including its sub-specialities were the top choices of students in terms of career preference in SA in earlier studies.^19,29^ Several specialities such as family medicine, ophthalmology, forensic medicine, public health medicine, ear, nose and throat, accident and emergency medicine were the least preferred choices in our study. This implies that substantial workforce shortages in such specialities may occur; thus, greater incentives are needed for graduating specialists in these fields towards meeting national needs.

Regarding sex differences in the choice of speciality, many studies have shown that males were more likely to select surgery,^14,20,22,25,26^ whereas females preferred internal medicine.^14,20,22^ Other studies found no difference between males and females in the choice of internal medicine as a speciality.^17,25^ Diderichsen et al found no statistically significant difference between the two groups; however, females have shown more interest in internal medicine than males, whereas the choice of surgery was equally distributed.^27^ In our study, males have shown more interest in both surgery and internal medicine than females, but the result was not statistically significant. Even though our study did not assess the reasons for sex inequality within the medical field, the most common reasons cited by many studies for the lack of female specialists in surgery include work-life balance, less procedural experience and inferior confidence in performing procedural skills, the perception that too much physical strength is required and the lack of mentorship in medical school.^27,30,31,32,33,34^ Other studies found that females experience gender discrimination and sexual harassment.^35,36^ Interestingly, studies show that at the end of the surgical clerkship, female students tend to express an increased interest in a surgical career compared to their male counterparts.^30^

Not surprisingly, in our study female medical students tend to opt more for obstetrics and gynaecology than males, even though the result was not statistically significant. The finding is similar to those reported in previous studies.^14,25,26^ In contrast, other studies reported that obstetrics and gynaecology were the most preferred speciality by males than females.^17,20,22^ Studies have shown that females preferred paediatrics as compared to males,^26,27^ while others found that males were more inclined towards paediatrics than females.^14^ In contrast, our findings showed no significant difference between males and females in the intention to pursue paediatrics as a speciality. Interestingly, in the present study, no males stated family medicine, public health medicine and forensic medicine, and no females stated accident and emergency medicine as the preferred speciality. Such specialities may need to intensify efforts to increase students’ interest and attract them.

Determining the main factors that influence speciality career choice is important because it provides insight into whether interest in speciality can be enhanced through increased educational exposure, illumination of existing misinformation or modification of current practice opportunities. Previous studies have noted various factors that influenced medical students to have a preference for a specific speciality.^16,17,24^ These factors included inspiration during clinical practice, financial reward, dedication to the field, benefits to a patient, the organisation of the department, possession of competency needed for the speciality, mentorship, friendliness and positive interpersonal relationships of lecturers in that field offered good advice and related well with students and personal interest. In SA, personal interests have been cited to be the most important factor influencing the choice of speciality.^19,29^ The current study did not evaluate the reasons medical students preferred certain speciality over others, but we believe that some of the factors mentioned may have contributed to the choice of specialities.

### Study limitations

This study has several limitations. Firstly, the study was conducted from a single academic institution; hence its findings are likely affected by the specific institutional curriculum, and therefore the findings cannot be generalised to other universities. Secondly, the study only analysed students’ preferences during the undergraduate programme, but not their actual choices. Thirdly, we did not assess the influencing factors why medical students prefer or do not prefer a specific speciality. Lastly, as it is a cross-sectional descriptive design, we cannot determine the potential influential relationships between characteristics of medical students and career choice and whether the participants would persist with their indicated choice and commit to it in the future.

## Conclusion

This study indicated that in our institution, students intend to pursue medical specialisation after graduation. We also observed no significant sex differences in one of the top three specialities preferences (surgery, obstetrics and gynaecology and internal medicine) – with both surgery and internal medicine male-dominated, while female students opt for obstetrics and gynaecology. It would be worth to explore factors that influence student speciality choices in our institution to deal with this imbalance. Also, inquire if there are any career guidance programmes available for students during their medical training period and examine the influence of career counsellors or advisors on speciality selection. Furthermore, policymakers should look into introducing the incentive schemes to deal with specialities that were least preferred.
